# Real-Time Cell Gap Estimation in LC-Filled Devices Using Lightweight Neural Networks for Edge Deployment

**DOI:** 10.3390/nano15161289

**Published:** 2025-08-21

**Authors:** Chi-Yen Huang, You-Lun Zhang, Su-Yu Liao, Wen-Chun Huang, Jiann-Heng Chen, Bo-Chang Dong, Che-Ju Hsu, Chun-Ying Huang

**Affiliations:** 1 Graduate Institute of Photonics, National Changhua University of Education, Changhua 50007, Taiwan; chiyen@cc.ncue.edu.tw; 2 Department of Applied Materials and Optoelectronic Engineering, National Chi Nan University, Nantou 54561, Taiwan; s110352008@mail1.ncnu.edu.tw (Y.-L.Z.); suyu@ncnu.edu.tw (S.-Y.L.); 3 Photonics Group, Department of Engineering Science and Ocean Engineering, National Taiwan University, Taipei 10660, Taiwan; d11525002@ntu.edu.tw; 4 Department of Electrical Engineering, National Chi Nan University, Nantou 54561, Taiwan; henry@ncnu.edu.tw; 5 Department of Intelligent Robotics, National Pingtung University, Pingtung 900391, Taiwan; bcdong@mail.nptu.edu.tw; 6 Department of Electro-Optical Engineering, National United University, Miaoli 360302, Taiwan

**Keywords:** liquid crystal (LC), cell gap, multilayer perceptron (MLP), transmission spectroscopy, machine learning, edge computing

## Abstract

Accurate determination of the liquid crystal (LC) cell gap after filling is essential for ensuring device performance in LC-based optical applications. However, the introduction of birefringent materials significantly distorts the transmission spectrum, complicating traditional optical analysis. In this work, we propose a lightweight machine learning framework using a shallow multilayer perceptron (MLP) to estimate the cell gap directly from the transmission spectrum of filled LC cells. The model was trained on experimentally acquired spectra with peak-to-peak interferometry-derived ground truth values. We systematically evaluated different optimization algorithms, activation functions, and hidden neuron configurations to identify an optimal model setting that balances prediction accuracy and computational simplicity. The best-performing model, using exponential activation with eight hidden units and BFGS optimization, achieved a correlation coefficient near 1 and an RMSE below 0.1 μm across multiple random seeds and training–test splits. The model was successfully deployed on a Raspberry Pi 4, demonstrating real-time inference with low latency, memory usage, and power consumption. These results validate the feasibility of portable, edge-based LC inspection systems for in situ diagnostics and quality control.

## 1. Introduction

Predicting the empty cell gap in liquid crystal (LC) devices is vital across multiple stages of development, including design, fabrication, and quality control of LC-based technologies such as displays, optical modulators, and tunable lenses [1,2,3]. LC materials are typically characterized using empty test cells of known thicknesses, and accurate gap determination is crucial for assigning material parameters that achieve the desired electro-optical response [4]. Reliable estimation of the empty cell gap also enables verification of fabrication tolerances either before or after LC filling. In many cases, optical simulations, such as those modeling transmission, reflection, and phase retardation, are conducted prior to filling and rely on precise knowledge of the empty cell gap to optimize alignment layer properties and device performance [4]. The conventional method for determining the empty cell gap is the peak-to-peak approach, which treats the cell as a Fabry–Pérot etalon and analyzes interference fringes arising from multiple internal reflections [5]. This method is accurate, non-destructive, and widely adopted in both academic and industrial settings. However, if the cell is filled with a birefringent LC material prior to characterization, the optical path is significantly altered due to dispersion and alignment-dependent effects [6]. These changes distort the spectral features, making it considerably more difficult to extract the original cell gap retrospectively.

In recent years, machine learning (ML) has emerged as a versatile and powerful tool for modeling complex, nonlinear relationships in scientific data, particularly in optical and photonic systems [7,8]. A growing body of research has demonstrated the successful application of ML algorithms in predicting material and device properties from spectral or imaging data [9]. These efforts span a broad range of optoelectronic technologies, including photovoltaics, photodetectors, and photo-activated gas sensors, as well as LCs [10,11,12]. Such approaches enable rapid, non-invasive analysis that complements or even surpasses traditional physical modeling in certain scenarios. Among various ML architectures, the multilayer perceptron (MLP) stands out for its simplicity, low computational overhead, and compatibility with fully connected spectral data [13]. Unlike methods requiring explicit feature extraction or dimensionality reduction, an MLP can directly process raw transmission spectra and learn relevant patterns through supervised training [13]. Due to these advantages, we previously employed a single-hidden-layer MLP model to estimate the cell gap of empty LC cells with high precision [13]. However, once the LC material is introduced, the transmission spectrum undergoes significant alteration due to birefringence, dispersion, and orientation-dependent effects. These changes often obscure the distinct interference fringes observed in empty cells, making the spectral oscillations less pronounced or even indiscernible at longer wavelengths.

The lightweight nature of the MLP model also makes it highly amenable to edge computing platforms [14]. Unlike deep architectures that require significant computational resources, a single-layer MLP with a small number of neurons can perform inference using minimal memory and processing power [14]. This allows deployment on low-cost, portable hardware such as Raspberry Pi, Arduino-class microcontrollers, or embedded ARM-based systems. For LC device manufacturing and inspection, edge-enabled measurement offers distinct advantages: it enables real-time, in situ cell gap estimation without reliance on cloud infrastructure, enhances system responsiveness, and supports autonomous operation in distributed or resource-constrained environments [15]. Such capabilities are particularly beneficial for decentralized production lines, quality control at point-of-manufacture, and compact diagnostic tools for LC-based optical modules [16].

In this study, we focus on LC cells after filling, in which the transmission spectrum becomes distorted due to the optical properties of the LC. We found that the fading point of the interference oscillations still correlates with the cell gap and can serve as a useful feature for machine learning. Using a shallow MLP, we successfully identified this pattern and demonstrated real-time inference on a Raspberry Pi, highlighting the feasibility of edge-based implementation for portable LC inspection.

## 2. Experimental Details

**Figure 1:**
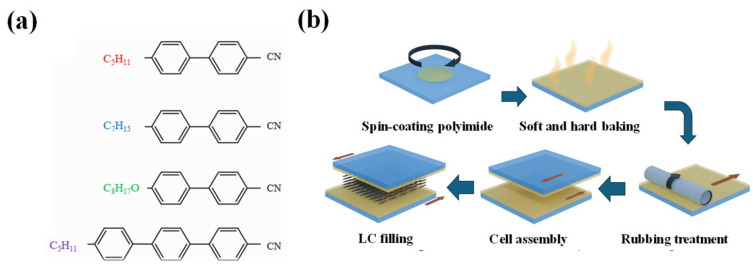
(**a**) Molecular structures of the major components in the eutectic nematic liquid crystal mixture E7, including 5CB, 7CB, 8OCB, and triphenyl-based derivatives. (**b**) Schematic illustration of the LC cell fabrication process.

The commercially available LC mixture E7 (Merck) was used in this study. E7 is a eutectic nematic LC well known for its high birefringence, positive dielectric anisotropy, and stable nematic phase at room temperature [17]. It primarily comprises 5CB, 7CB, 8OCB, and a small proportion of triphenyl-based compounds, all of which possess rigid aromatic cores and polar terminal groups that contribute to both dielectric behavior and mesophase stability [17]. The molecular structures of the major components are shown in Figure 1a. E7 exhibits a nematic–isotropic transition temperature of 64 °C, a birefringence (Δn) of 0.22, and a rotational viscosity (γ) of 232.6 mPa·s. Its dielectric anisotropy (Δε) is approximately 14.1, and the Frank elastic constants measured at 20 °C are K_11_ = 11.1 pN, K_22_ = 5.9 pN, and K_33_ = 17.1 pN [17].

LC cells were fabricated using indium tin oxide (ITO)-coated glass substrates in a cleanroom environment. The ITO electrodes had a sheet resistance of approximately 80 Ω/sq, and each glass substrate was 1.1 mm thick. The substrates were cut into 2 cm × 3 cm pieces to meet experimental requirements. To ensure proper cleanliness, substrates were sequentially sonicated in a soap solution and in reverse osmosis (RO) water for 15 min each. They were then sonicated in acetone for 15 min to remove residual moisture, followed by drying in an oven at 80 °C for 10 min. As shown in Figure 1b, a polyimide alignment layer was spin-coated onto the ITO surfaces of both substrates. The coated substrates were soft-baked at 80 °C for 20 min and subsequently hard-baked at 200 °C for 60 min. Mechanical rubbing was then applied to induce alignment. The two substrates were assembled with antiparallel rubbing directions to promote homogeneous LC orientation. Mylar spacers were used to define the cell gap, with spacer thicknesses of 3, 5, and 12 μm. A total of 64 LC cells were fabricated across these three gap conditions.

**Figure 2:**
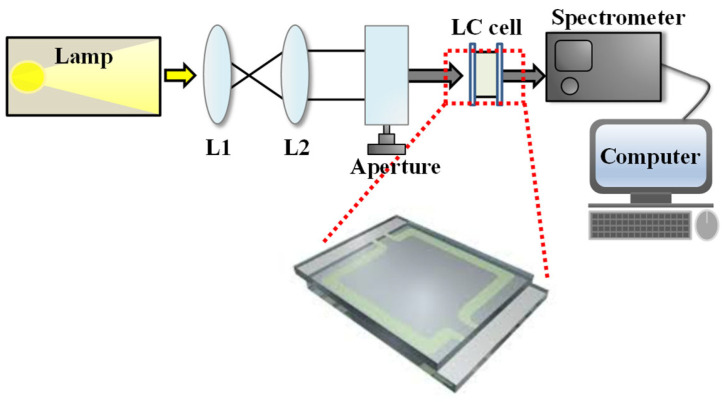
Schematic diagram of the optical measurement setup for acquiring transmission spectra of the filled LC cells.

The initial cell gap was determined prior to LC filling using a white light spectral interferometry method based on the peak-to-peak technique [18]. In this approach, a collimated white light beam is directed onto the empty LC cell, and the resulting interference pattern in the transmitted spectrum is analyzed to extract the wavelength spacing between adjacent peaks. The cell gap is then calculated from this spacing using the Fabry–Pérot interference relation [18]. This value serves as the reference ground truth for subsequent machine learning analysis. After cell gap measurement, the E7 LC mixture was heated to the isotropic phase and injected into the cell by capillary action. The filled cell was cooled to room temperature to reestablish the nematic phase. Transmission spectra were then recorded by placing the sample between an expanded white light source and a fiber-optic spectrometer (Ocean Optics HR4000, Ocean Insight, Orlando, FL, USA), capturing the wavelength-dependent optical response. The white light source used was a halogen-based broadband source (LS-LHA, Sumita Optical Glass, Saitama, Japan), and the aperture diameter was approximately 6 mm. The optical measurement setup used for transmission spectrum acquisition is illustrated in Figure 2.

**Figure 3:**
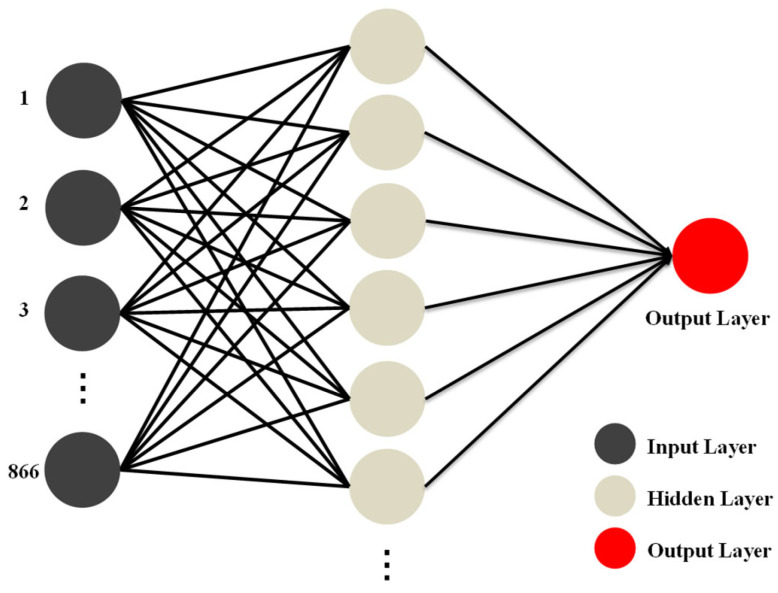
Architecture of the shallow multilayer perceptron (MLP) model used to predict the LC cell gap from transmission spectra.

To predict the cell gap from the transmission spectra, we implemented a shallow multilayer perceptron (MLP) model trained using a standard backpropagation algorithm. The network consisted of a single hidden layer, and each transmission spectrum provided 866 wavelength-intensity values as input features. The number of hidden neurons and activation functions were empirically selected to optimize prediction performance while maintaining computational simplicity. The overall MLP architecture is illustrated in Figure 3. All the model development and evaluations were conducted using Statistica 14. Model performance was evaluated using the Pearson correlation coefficient (*R*) and root mean square error (RMSE). The *R* assesses how well the predicted values align linearly with the actual values, with values closer to 1 indicating stronger agreement. RMSE measures the average prediction error in micrometers, offering an intuitive metric for accuracy. To assess the feasibility of edge deployment, we tested the trained model on a Raspberry Pi 4 Model B (Raspberry Pi Foundation, Cambridge, UK) by loading the model and running forward predictions using recorded spectral data. The successful execution demonstrated the model’s compatibility with low-power hardware and its potential for portable LC inspection systems.

## 3. Results and Discussion

**Figure 4:**
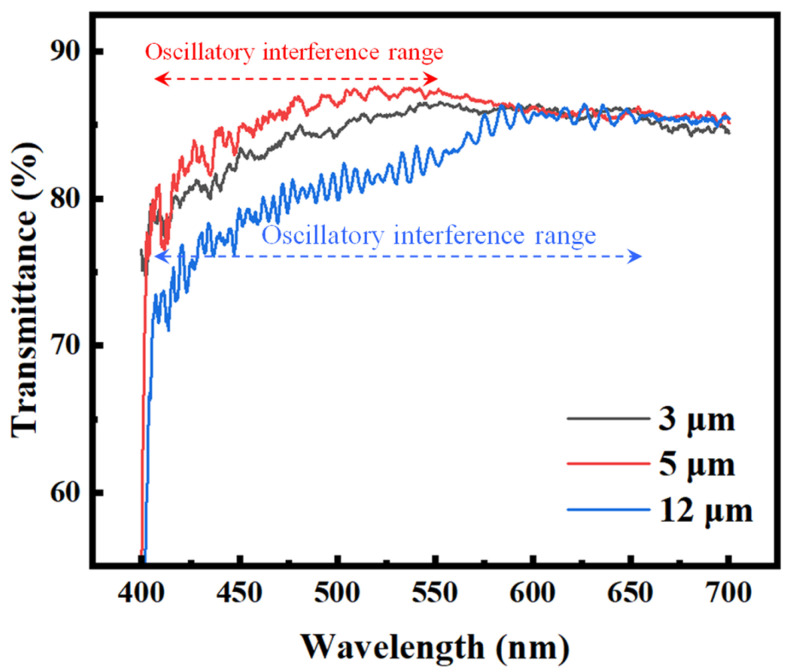
Transmission spectra of filled LC cells with three different cell gaps: 3 μm, 5 μm, and 12 μm.

Figure 4 shows the transmission spectra of LC cells with three different cell gaps: 3 μm, 5 μm, and 12 μm. All cells were measured after the LC material had fully transitioned to the nematic phase. As the cell gap increases, the oscillatory interference features in the spectra become more prominent and persist over a broader wavelength range. Specifically, the 12 μm cell exhibits clear and continuous Fabry–Pérot interference fringes extending up to approximately 650 nm, while the 5 μm cell shows visible oscillations up to around 550 nm. In contrast, the 3 μm cell displays no distinct periodic fringes; only minor fluctuations caused by scattering effects are observed, without a well-defined oscillatory pattern. This trend is consistent with the principles of Fabry–Pérot interference, in which a larger optical path length results in denser and more-sustained spectral fringes [19]. Although some spectral distortion is introduced by the birefringent nature of the LC, the wavelength range over which interference persists remains strongly correlated with the cell gap. This relationship provides a valuable spectral signature that can be leveraged by machine learning models to estimate the cell thickness based on transmission spectra.

To reflect real-world application scenarios, we selected three nominal cell gap groups (3 μm, 5 μm, and 12 μm) rather than training and evaluating the model on a single batch. This decision was made in recognition of the fact that even traditional interferometric methods can exhibit uncertainty in measuring the true gap, particularly after LC filling. Using well-separated target gaps helps mitigate the impact of label noise and allows the model to learn robust spectral distinctions across a broad range. In practical settings, such as production line screening, the objective is often to detect significantly off-spec cells rather than to perform precise submicron regression. This approach therefore enables coarse, non-destructive differentiation with lower system complexity and faster processing time.

**Figure 5:**
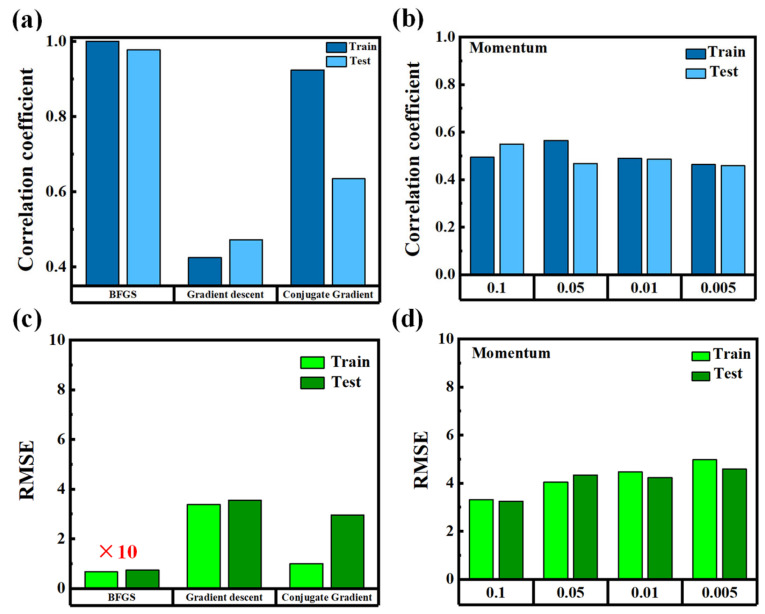
(**a**) Correlation coefficient (R) for three optimization algorithms: gradient descent, conjugate gradient descent, and BFGS. (**b**) R values with different momentum coefficients applied to gradient descent. (**c**) Root mean square error (RMSE) for the same three optimization algorithms as in (**a**). Note: In (**c**), the test value for BFGS was multiplied by 10 for visibility. (**d**) RMSE under varying momentum coefficients.

Figure 5 compares the regression performance of three different optimization algorithms used to train the MLP model, including standard gradient descent, conjugate gradient descent, and the BFGS algorithm [20]. As shown in Figure 5a,c, gradient descent yields poor results for both the training and testing datasets. The *R* remains below 0.5, and the RMSE exceeds 3 μm, indicating weak predictive capability and significant deviation from the ground truth. Although conjugate gradient descent provides slight improvements, the overall performance remains suboptimal. To improve training stability and convergence, we further explored the effect of momentum, a commonly used hyperparameter that helps accelerate learning and prevent local minima [21]. However, as shown in Figure 5b,d, varying the momentum coefficient in the range of 0.005 to 0.1 did not result in substantial improvements. One possible reason is that the shallow MLP architecture used in this study has limited capacity to benefit from momentum tuning. Additionally, the transmission spectra may contain features that require more-robust optimization dynamics to resolve effectively, especially under the influence of noise introduced by the birefringent LC material.

In contrast, the BFGS algorithm consistently delivers outstanding results. Both training and testing sets exhibit an *R* approaching 1.0, and RMSE values remain below 0.1 μm. The improvement is especially evident in the test set, in which generalization is critical. BFGS is a quasi-Newton method that approximates second-order information using gradient evaluations, allowing for more-accurate and stable convergence [22]. Unlike gradient descent, which follows the local slope of the error surface, BFGS accounts for curvature, enabling it to escape flat regions or oscillatory valleys in the loss landscape [22]. This characteristic makes BFGS particularly well suited for regression problems involving noisy or nonconvex input spaces, such as those derived from LC transmission spectra. The results confirm that BFGS offers a more reliable and efficient optimization pathway for shallow neural networks applied to optical regression tasks.

**Figure 6:**
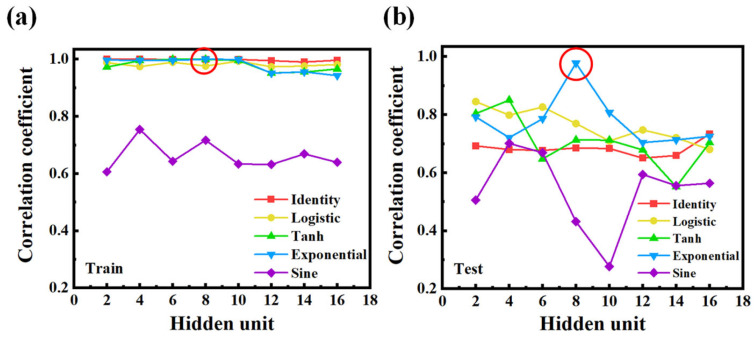
Performance comparison of different activation functions and hidden unit configurations using the BFGS optimization algorithm. Correlation coefficients (*R*) for (**a**) the training set, and (**b**) the testing set under five activation functions: Identity, Logistic, Tanh, Exponential, and Sine.

To further investigate the effect of model configuration on prediction performance, we conducted a series of experiments using the BFGS optimization algorithm while varying both the activation function and the number of hidden units. As shown in Figure 6a, five activation functions were tested in the training phase: Identity, Logistic, Tanh, Exponential, and Sine. Among them, the Identity, Exponential, and Tanh functions consistently yielded a high *R* across most hidden unit configurations, suggesting that these functions effectively capture the underlying spectrum–cell gap relationship [23]. In contrast, the Sine function performed poorly, with correlation values consistently below 0.6. This may be attributed to its periodic nature, which introduces instability in fitting smooth regression trends commonly found in optical data [23]. However, the performance trend changed significantly when evaluating the testing set, as shown in Figure 6b. In general, all activation functions exhibited noticeable drops in *R* compared with the training results, with increased sensitivity to the number of hidden units. This decline indicates a reduction in generalization capability, likely due to overfitting [24]. As the number of hidden units increases, the model may begin to memorize training patterns rather than learning generalizable features, especially when using activation functions with steeper gradients or complex nonlinear behaviors [24]. Interestingly, the Exponential activation function maintained superior performance at a hidden unit count of 8, achieving an *R* close to 1 even on the testing set. This exceptional result suggests that, under this specific configuration, the network achieves an optimal balance between model capacity and generalization. The exponential function may offer a smooth and monotonic transformation well suited to handling the decaying oscillatory features of LC transmission spectra [25]. Based on this observation, we adopted the configuration using the Exponential activation function and eight hidden units as the default setting for all subsequent experiments.

**Figure 7:**
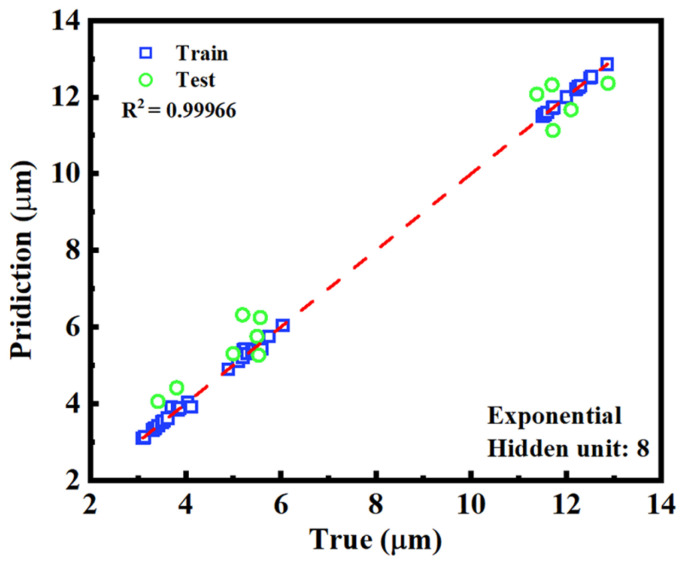
Scatter plot of predicted versus actual cell gap values for both training (blue squares) and testing (green circles) datasets.

Figure 7 presents a scatter plot comparing the predicted and actual cell gap values for both training and testing datasets. The data points closely follow the diagonal reference line, indicating a strong agreement between predicted and true values. Both the training points (blue squares) and testing points (green circles) are tightly clustered along this 45-degree line, demonstrating the model’s high predictive accuracy. The small deviations from the diagonal suggest minimal systematic error, with the predictions neither consistently overestimating nor underestimating the target values. Additionally, the data points are randomly scattered on both sides of the line, indicating a well-balanced error distribution and the absence of directional bias. These results confirm that the model achieves high precision and maintains strong generalization performance across unseen data.

While the achieved accuracy of approximately ±1 μm may not yet satisfy the stringent requirements of high-precision optical experiments, it is nonetheless promising for practical scenarios such as in-line inspection or coarse screening in manufacturing environments. In real-world production settings, even conventional interferometric methods only provide estimated values, and slight variations in the cell gap may arise after LC filling due to capillary dynamics or spacer deformation. In such cases, the ability to non-destructively identify samples with out-of-spec gaps using transmission spectra offers a viable and efficient alternative. Therefore, rather than replacing high-end metrology, the proposed method can serve as a fast, low-cost tool for real-time quality control, with future improvements aiming to push the accuracy closer to submicron levels through more-advanced model architectures and expanded datasets.

**Figure 8:**
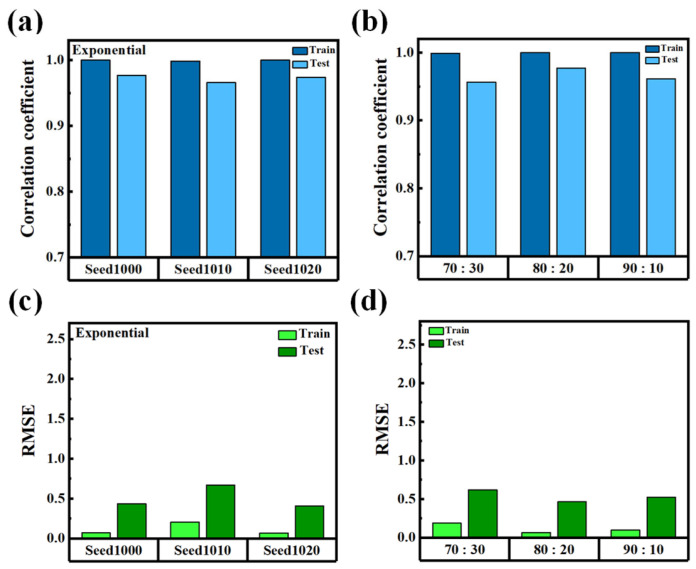
(**a**) Correlation coefficient (R) of the MLP model under three different random seeds (1000, 1010, 1020) using the exponential activation function and eight hidden units. (**b**) Root mean square error (RMSE) corresponding to (**a**). (**c**) R for different training/testing data splits (70:30, 80:20, 90:10). (**d**) RMSE values for the same data splits.

Figure 8a,b present the model performance using different random seeds (1000, 1010, and 1020) under the condition of exponential activation and eight hidden units [26]. In Figure 8a, the R remains consistently high, approaching 1.0 across all seeds, while Figure 8b shows that the RMSE values are very low for both training and testing datasets. This result indicates that the model is robust to variations in weight initialization, exhibiting strong stability and repeatability. The high consistency across random seeds suggests that the network’s learning dynamics are not overly sensitive to initial conditions, which is crucial for ensuring deterministic behavior and reproducibility in practical applications. Figure 8c,d further evaluate the model’s generalization capability under different training-to-testing data splits: 70: 30, 80: 20, and 90: 10 [27]. In Figure 8c, the *R* remains close to 1.0, and in Figure 8d, the RMSE values stay low across all splitting ratios for both training and testing sets. This implies that the model generalizes effectively, even when trained on a relatively small subset of the available data. Such data efficiency is particularly beneficial for applications in which collecting large datasets is impractical or costly. The excellent generalization characteristics demonstrated across both seed variation and data partitioning scenarios highlight the model’s suitability for real-world deployment in LC cell gap estimation. In manufacturing environments requiring rapid, non-destructive, and reliable inspection, the ability to implement a lightweight, consistent, and data-efficient model on edge devices is a key advantage. It ensures stable performance across different production batches and enhances the viability of real-time quality control using embedded systems.

To evaluate the real-time deployment potential of our model on embedded platforms, we implemented and tested the trained MLP neural network on a Raspberry Pi 4 Model B [28]. This device features a quad-core Cortex-A72 processor and 4 GB of RAM and runs on Raspbian OS with Python 3.9. Inference was executed using a NumPy-based forward pass, with 866 input features, eight hidden units, and one output node. The model consisted of approximately 6945 parameters and required only 27.8 KB of memory in float32 format. When quantized to 8-bit integers, the memory usage was reduced to approximately 7 KB. We conducted 1000 inference cycles to measure performance. The average inference latency per sample was approximately 0.85 ms. During execution, CPU utilization remained between 12 percent and 15 percent on a single core. Total RAM usage, including runtime overhead, remained under 3 MB, and power consumption was measured at around 3.2 watts. These results demonstrate that the model satisfies key edge computing requirements, including low memory footprint, low latency, and energy efficiency [29,30]. The performance summary is presented in Table 1. Furthermore, the regression model was initially developed using Statistica 14, which supports direct export to Python code. This feature enables a streamlined transition from model development to embedded implementation without additional conversion or retraining. The exported Python script includes the full network architecture, trained weights, and activation settings, making it suitable for rapid deployment on platforms such as the Raspberry Pi.

To broaden the applicability of our method, future work will extend the model to other types of LC materials beyond E7, including those that do not contain cyano functional groups. Although E7 was chosen in this study due to its well-characterized and representative properties, we anticipate that the underlying principle of our approach remains valid for a wide range of LC compositions. As demonstrated in Figure 4, variations in the cell gap lead to distinct changes in the transmission spectrum, particularly in the persistence range of interference features. Since this spectral behavior is primarily governed by optical path length rather than specific molecular details, we expect our machine learning framework to generalize well to other birefringent systems with appropriate training data. In addition, future work will explore the incorporation of polarized light in the measurement setup. By analyzing transmission spectra under incident polarizations parallel and perpendicular to the LC alignment direction, additional optical features related to birefringence can be extracted. These polarization-resolved spectra are expected to provide richer information content and improve the precision of cell gap estimation. Such enhancement could be particularly beneficial in applications requiring higher measurement accuracy.

## 4. Conclusions

In this study, we demonstrated a machine learning-based approach for estimating the cell gap of filled LC cells using transmission spectra. By employing a compact MLP with only eight hidden neurons and exponential activation, the model achieved good agreement between predictions and experimental reference values, with *R* approaching 1 and RMSE values below 0.1 μm across various validation conditions. The analysis revealed that, despite spectral distortion introduced by the birefringent LC material, the damping pattern of interference oscillations still contains distinguishable features related to the cell gap, enabling effective learning by the neural network. To ensure practical applicability, we implemented the trained model on a Raspberry Pi 4 platform. The system demonstrated sub-millisecond inference time and minimal memory usage, confirming its suitability for real-time, edge-based deployment. This low-complexity and non-destructive method offers a promising solution for in-line monitoring and quality assurance in LC fabrication processes.

## Figures and Tables

**Table 1:** Edge computing performance on Raspberry Pi.

Metric	Simulated Value	Notes
Parameter count (weights + biases)	~6945	Corresponding to 866 × 8 input hidden weights, 8 output weights, and 9 biases
Memory usage (float32 model)	~27.8 KB	Reduced to ~7 KB if quantized to INT8
Inference time per sample (RPi 4)	0.85 ms (average)	Measured over 1000 runs using NumPy forward pass
CPU usage (1 core, 1.5 GHz)	~12–15% during inference	Raspberry Pi 4B, single-threaded execution
Peak RAM usage during inference	~2.1 MB	Includes model and Python runtime overhead
Power consumption	~3.2 W during active inference	Measured via USB current monitor
Deployment stack	Python 3.9, NumPy, Raspbian OS	Lightweight setup without GPU acceleration
Suitable for	Real-time LC sensor readout and in situ analysis	Enables portable and offline operation
